# Fenofibrate increases very-long-chain sphingolipids and improves blood glucose homeostasis in NOD mice

**DOI:** 10.1007/s00125-019-04973-z

**Published:** 2019-08-13

**Authors:** Laurits J. Holm, Martin Haupt-Jorgensen, Jano D. Giacobini, Jane P. Hasselby, Mesut Bilgin, Karsten Buschard

**Affiliations:** 1 The Bartholin Institute, Department of Pathology, Rigshospitalet, Copenhagen Biocenter, Ole Maaløes Vej 5, 2200 Copenhagen N, Denmark; 2 Cell Death and Metabolism Unit, Center for Autophagy, Recycling and Disease, Danish Cancer Society Research Center, Copenhagen, Denmark; 3 Department of Pathology, Copenhagen University Hospital, Rigshospitalet, Copenhagen, Denmark

**Keywords:** Blood glucose homeostasis, Ceramide, Fenofibrate, Glycerophospholipid, NOD mice, Sphingolipid, Sulfatide, Sympathetic nerve fibres, Type 1 diabetes, Tyrosine hydroxylase

## Abstract

**Aims/hypothesis:**

Sphingolipid metabolism regulates beta cell biology and inflammation and is abnormal at the onset of type 1 diabetes. Fenofibrate, a regulator of sphingolipid metabolism, is known to prevent diabetes in NOD mice. Here, we aimed to investigate the effects of fenofibrate on the pancreatic lipidome, pancreas morphology, pancreatic sympathetic nerves and blood glucose homeostasis in NOD mice.

**Methods:**

We treated female NOD mice with fenofibrate from 3 weeks of age. The pancreatic lipidome was analysed using MS. Analysis of pancreas and islet volume was performed by stereology. Islet sympathetic nerve fibre volume was evaluated using tyrosine hydroxylase staining. The effect on blood glucose homeostasis was assessed by measuring non-fasting blood glucose from age 12 to 30 weeks. Furthermore, we measured glucose tolerance, fasting insulin and glucagon levels, and insulin tolerance.

**Results:**

We found that fenofibrate selectively increases the amount of very-long-chain sphingolipids in the pancreas of NOD mice. In addition, we found that fenofibrate causes a remodelling of the pancreatic lipidome with an increased amount of lysoglycerophospholipids. Fenofibrate did not affect islet or pancreas volume, but led to a higher volume of islet sympathetic nerve fibres and tyrosine hydroxylase-positive cells. Fenofibrate-treated NOD mice had a more stable blood glucose, which was associated with reduced non-fasting and increased fasting blood glucose. Furthermore, fenofibrate improved glucose tolerance, reduced fasting glucagon levels and prevented fasting hyperinsulinaemia.

**Conclusions/interpretation:**

These data indicate that fenofibrate alters the pancreatic lipidome to a more anti-inflammatory and anti-apoptotic state. The beneficial effects on islet sympathetic nerve fibres and blood glucose homeostasis indicate that fenofibrate could be used as a therapeutic approach to improve blood glucose homeostasis and prevent diabetes-associated pathologies.

**Electronic supplementary material:**

The online version of this article (10.1007/s00125-019-04973-z) contains peer-reviewed but unedited supplementary material, which is available to authorised users.

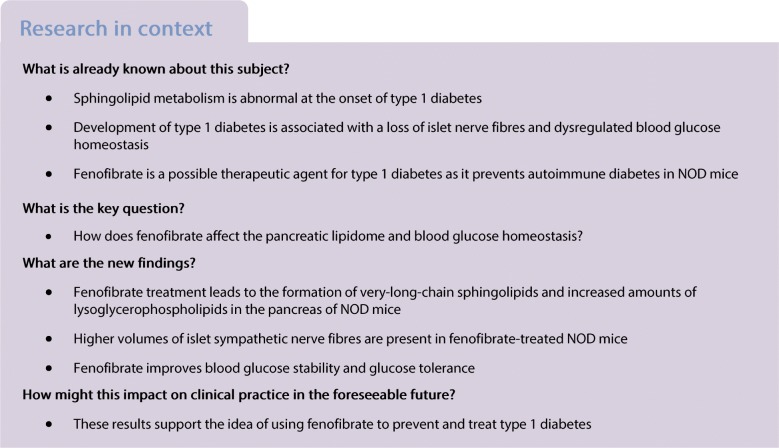



## Introduction

Experimental studies have demonstrated the importance of sphingolipids in regulating beta cell biology and inflammation [1, 2]. The central sphingolipid metabolite is ceramide, which is generally considered to induce apoptosis and insulin resistance [3]. Diversity in sphingolipid structure is accomplished by adding headgroups to the hydroxyl group of ceramide and by varying the length of the acyl chain (C14–C26) [4]. Adding a phosphocholine generates sphingomyelin, which is a major component of myelin sheaths [5]. The addition of galactose and sulfate to ceramide forms sulfatide, which is an insulin chaperone and regulator of insulin secretion [6, 7]. Furthermore, C24 sulfatide is involved in regulating natural killer T cells by CD1d presentation [8]. Sphingolipids are also of interest as a major component of the myelin sheath surrounding sympathetic nerves [9]. Sympathetic nerves are involved in the pathology of type 1 diabetes, with an observed loss of islet sympathetic nerve fibres in individuals with newly diagnosed type 1 diabetes [10] and NOD mice [11]. Sympathetic neurons inhibit insulin and stimulate glucagon secretion to prevent hypoglycaemia [12, 13]. The loss of pancreatic sympathetic nerve fibres is, therefore, a contributing factor to the early loss of glucagon secretion observed in adolescents with type 1 diabetes and NOD mice [11, 14].

Another lipid class is the glycerophospholipids (GPLs), which make up more than 50% of the lipids in plasma membranes [15]. GPLs and their derivate lysoGPLs (lacking one or both lipid chains) are important structural components that are known to regulate cell biology and inflammation [15]; however, they are relatively undescribed in relation to type 1 diabetes.

We have previously shown that sphingolipid metabolism is abnormal at the onset of type 1 diabetes, as evidenced by a reduced islet level of sulfatide and reduced expression of several enzymes involved in sphingolipid metabolism [16]. Furthermore, polymorphisms in genes involved in sphingolipid metabolism are linked to the genetic predisposition to type 1 diabetes and to the degree of cellular islet autoimmunity. In this regard, we have previously reported that fenofibrate, a peroxisome proliferator-activated receptor alpha agonist, increased islet sulfatide levels and completely prevented diabetes in NOD mice, thereby making fenofibrate a possible new therapy for type 1 diabetes [16].

Additional insight into the mechanisms of action of fenofibrate is needed to better determine its therapeutic potential. Therefore, the aims of this study were to examine the effects of fenofibrate on pancreas morphology and the lipidome, and to evaluate its effects on blood glucose homeostasis.

## Methods

### Animals

Animal experiments were conducted in agreement with Directive 2010/63/EU of the European Parliament and of the Council of 22 September 2010 on the protection of animals used for scientific purposes, and the Danish Animal Experimentation Act (LBK 474 15/05/2014). The Danish Animal Experiments Inspectorate (reference 2016-15-0201-00841) and the local ethical committee (EMED: P 15-383, P 16-440 and P 18-408) approved the study.

Female NOD mice (Taconic Biosciences, Hudson, NY, USA) were housed at the Department of Experimental Medicine (University of Copenhagen, Copenhagen, Denmark) and kept in a specific pathogen-free animal facility (temperature 22°C, 12 h light cycle, air changed 16 times per hour and humidity 55 ± 10%). Three-week-old mice were randomly distributed into two groups receiving either a standard Altromin 1320 diet (Altromin, Lage, Germany) or a modified Altromin 1320 diet containing 0.1% fenofibrate (Sigma, St Louis, MO, USA). All mice had free access to food and drinking water. There was no difference in body weight between the two groups at the start of the experiment. Mice were treated from the age of 3 weeks and until the experiment was terminated. Diabetic mice were excluded from the study. Diabetes diagnosis was based on two blood glucose measurements >12 mmol/l with an interval of 2 days. Mice were weighed each week, and food and water intake were measured weekly by weighing the food racks and water flask, respectively. Glucose monitoring was performed using FreeStyle Lite (Abbott Diabetes Care, Alameda, CA, USA) by measurement from the tail tip. Mice were put into individual cages and fasted for 6 h from 08:00. Fasting serum insulin and glucagon levels were measured using a Mercodia mouse insulin or glucagon ELISA kit, respectively (Mercodia, Uppsala, Sweden). Fasting insulin above 1000 pmol/l was defined as hyperinsulinaemia.

### ITT and GTT

Thirteen-week-old mice were fasted as described above. Fasting blood glucose was measured from the tail tip and used as time point 0 min. Mice were then, for the GTT, i.p. injected with 0.01 ml of 1 mol/l glucose per g body weight. Glucose concentrations were measured as described above at time 15, 30, 45, 60, 90 and 120 min. Because of the difference in fasted blood glucose between the groups, the results are shown as the per cent increase in blood glucose compared with the fasting blood glucose concentration of each mouse. AUC was calculated using the fasting blood glucose level as baseline. For the ITT, mice were i.p. injected with 0.75 U insulin (Actrapid, Novo Nordisk, Bagsværd, Denmark) per kg body weight. Glucose concentrations were measured at time 15, 30, 45, 60, 90, 120 and 150 min. Results are shown as the per cent change in blood glucose compared with the fasting blood glucose concentration of each mouse. Glucose concentrations declined for the first 60 min. An AUC for time 0–60 min was used as a measurement of insulin tolerance. Glucose concentrations increased during the 60–150 min period, and an AUC calculated with 60 min glucose as 100% was used to measure the mice’s response to hypoglycaemia.

### Organ weight, insulitis and stereology

The mice were killed and their organs were carefully removed and weighed. Pancreases were fixed in 10% neutral buffered formalin overnight until paraffin embedding. Pancreases were cut in 5 μm sections in their entirety. For insulitis scoring, sections were stained with H&E and evaluated using a BX53 microscope (Olympus America, Melville, NY, USA). A total of 30 islets from each mouse were scored blindly according to the following scale: 0, no infiltration; 1, intact islets but with few mononuclear cells surrounding the islets; 2, peri-insulitis (multiple mononuclear cells surrounding the islets); 3, islet infiltration below 50%; 4, islet infiltration above 50%. For images of the different insulitis scores, see [17]. For stereological evaluation of islet and pancreas volume, every 20th section was H&E stained and scanned at ×10 magnification using NanoZoomer- XR (Hamamatsu, Hamamatsu City, Japan). The resulting images were investigated using newCAST software version 2019.02. (Visiopharm, Hoersholm, Denmark). A point-counting grid with 25 intersections and 1 encircled unit intersections was applied to the images, and they were counted blindly for the total number of intersections touching islets ∑P(islet) and the total number of unit intersections touching pancreases ∑P(pancreas).

For stereology analysis of sympathetic nerves, every 30th section was stained for tyrosine hydroxylase (TH; ab112, Abcam, Cambridge, UK; diluted 1:150) and H&E. Slides were scanned at ×40 magnification and a point-counting grid with 256 intersections was applied to the images, and they were counted blindly for the total number of intersections touching a TH-positive area ∑P(TH). TH staining was divided into two fractions, fibres and cells. Islet, pancreas or TH volume was estimated using the following equation based on the Cavalieri method [18].

$$ V=T\times a/p\times \sum P\left( islet, pancreas, or\  TH\right) $$

Where *V* is the volume, *a/p* designates the area per point of the grid and *T* specifies the section distance.

### Immunofluorescence

Pancreatic tissues were fixed as described above and cut in 1.5 μm sections. Following deparaffinisation and rehydration, the sections were rinsed in PBS and treated with target retrieval solution citrate pH 6.1 (Dako, Ely, UK) for 30 min at 99°C, then cooled to room temperature. Following a 5 min rinse in PBS, the sections were blocked in 2% BSA and 0.05% Tween 20 in PBS for 30 min at room temperature. Guinea pig anti-insulin (ab195956, diluted 1:800), rabbit anti-glucagon (ab92517, diluted 1:400) and sheep anti-TH (ab113, diluted 1:25) were applied for 1 h at room temperature (insulin), 24 h at 4°C (glucagon) or 48 h at 4°C (TH). The sections were then rinsed twice in PBS and once in blocking buffer, each for 5 min. Relevant secondary antibodies labelled with Cy3 (insulin, ab102370, diluted 1:100), phycoerythrin (glucagon, ab72465, diluted 1:100) or FITC (TH, ab6896, diluted 1:1000) were applied for 1 h at room temperature. All antibodies were from Abcam. The sections were washed three times, each for 5 min, mounted in Prolong Gold Antifade with DAPI (Life Technologies, Carlsbad, CA, USA) and examined using a Zeiss LM710 confocal microscope (Zeiss, Oberkochen, Germany). For quantification, the slides were scanned using a Zeiss Axio Scan.Z1 (Zeiss) and ten islets were chosen randomly from each mouse (*n* = 6). For every islet, the number of cells co-expressing hydroxylase and glucagon/insulin were counted.

### Lipid measurement

For lipid measurement, 10 mg samples were taken from the tail of the pancreas, snap frozen and kept at −80°C until analysis. Samples were homogenised at 4°C on TissueLyser II (QIAGEN, Hilden, Germany) in 155 mmol/l ammonium bicarbonate. Total protein concentration was measured using the Pierce BCA Protein Assay (Thermo Fisher Scientific, Waltham, MA, USA). Aliquots corresponding to 100 μg protein were subjected to lipid extraction by a modified Bligh and Dyer protocol executed at room temperature [19]. The sample aliquots were spiked with 10 μl of 50 nmol (corresponding to 0.5 pmol) of SHexCer 30:1:2 standard. Quantification of sulfatide species was performed on a (U)HPLC UltiMate 3000 RSLCnano System (Thermo Fisher Scientific) interfaced online to the Q-Exactive Quadrupole-Orbitrap Mass Spectrometer (Thermo Fisher Scientific). We used a silica column 0.5 × 150 mm (YMC-Pack Silica analytical column, 3 μm particles). We used LipidXplorer version 1.2.7 (https://wiki.mpi-cbg.de/lipidx/Main_Page) to extract data, selecting a time range based on the eluted peaks of the sulfatide species. For the quantitative shotgun lipidomics analysis, aliquots corresponding to 20 μg of protein were subjected to lipid extraction by a modified two-step Bligh and Dyer protocol executed on ice [20, 21]. The aliquots of tissue homogenate were spiked with 15 μl internal lipid standard mix containing 30 pmol cholesteryl ester 15:0-D7, 20 pmol ceramide 18:1;2/12:0;0, 10 pmol diacylglycerol 12:0/12:0, 20 pmol dihexose ceramide 18:1;2/12:0;0, 25 pmol hexose ceramide 18:1;2/12:0;0, 25 pmol lysophosphatic acid 17:0, 20 pmol lysophosphatidylcholine (LPC) 10:0, 25 pmol lysophosphatidylethanolamine 13:0, 15 pmol lysophosphatidylglycerol 17:1, 20 pmol lysophosphatidylinositol 13:0, 20 pmol lysophosphatidylserine 17:1, 25 pmol phosphatidic acid 12:0/12:0, 20 pmol phosphatidylcholine (PC) ether 18:1/18:1, 25 pmol PE 12:0/12:0, 15 pmol phosphatidylglycerol 12:0/12:0, 15 pmol phosphatidylinositol 8:0/8:0, 20 pmol phosphatidylserine 12:0/12:0 and 20 pmol sphingomyeline 18:1;2/12:0;0. The combined two-step lipid extracts were subjected to MS analysis using a Q-Exactive Quadrupole-Orbitrap Mass Spectrometer equipped with a TriVersa NanoMate (Advion Biosciences, Ithaca, NY, USA). Data processing was performed using LipidXplorer. All solvents were HPLC-grade for lipid extraction and LC-MS grade for LC-MS analysis. Methanol, water and chloroform for lipid extraction were supplied by Rathburn (Walkerburn, UK). Water, methanol and acetonitrile for LC-MS were supplied by VWR (Radnor, PA, USA). Ammonium bicarbonate was supplied by Sigma Aldrich (Darmstadt, Germany).

### Statistics

Principal component analysis was performed using R statistics software version 3.4.0 (R Development Core Team, Vienna, Austria) and a *p* value of 0.01. The remaining statistical analyses were performed using GraphPad Prism version 7.0 (La Jolla, CA, USA). All data were assessed to ensure equal variance and normal distribution between groups. For comparisons between groups, a two-tailed unpaired Student’s *t* test was used. To identify significantly altered lipid classes a false discovery rate of 5% was used, as determined by the two-stage linear step-up procedure of Benjamini, Krieger and Yekutieli [22]. Data were log-transformed before analysis if not normally distributed. If this did not resolve the normality problem then a Mann–Whitney *U* test was performed. Correlation analysis between neuronal volume and the insulitis score was performed using linear regression. The percentage of mice with hyperinsulinaemia was evaluated using a *χ*^2^ test. A *p* value of less than 0.05 was considered significant. Data are shown as means ± SD when a two-tailed unpaired Student’s *t* test was used and as medians (interquartile range) when a Mann–Whitney *U* test was performed. *n* denotes the number of animals used.

## Results

### Fenofibrate promotes the formation of very-long-chain sphingolipids

**Fig. 1:**
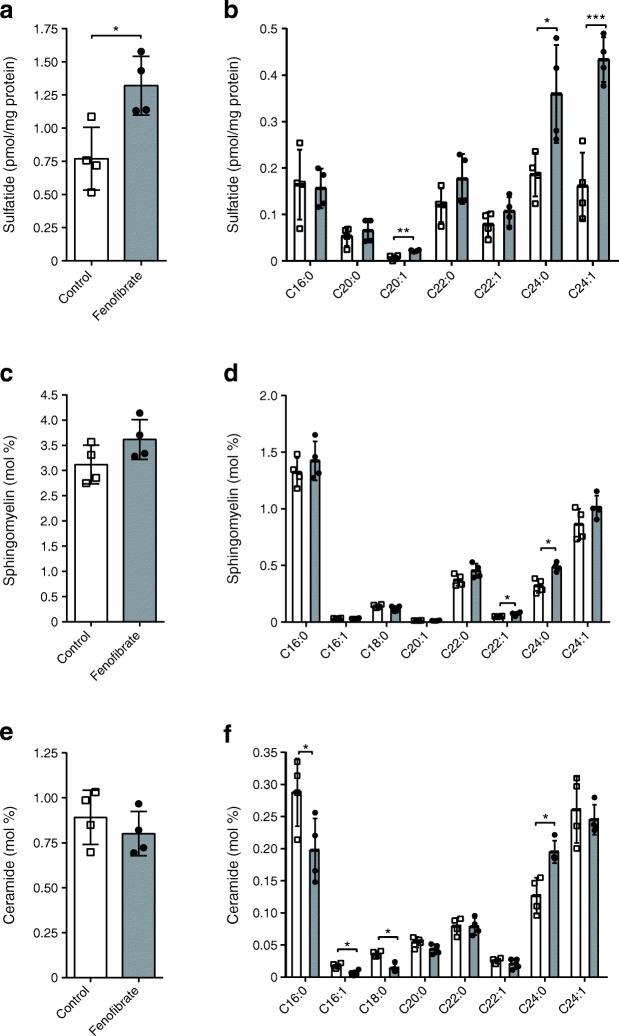
Fenofibrate promotes the formation of very-long-chain sphingolipids. Pancreatic samples taken from 13-week-old mice were analysed by LC-MS to evaluate (**a**) total sulfatide content and (**b**) the amounts of individual sulfatide species (as pmol/mg protein). (**c**–**f**) The remaining lipidome was analysed using a shotgun lipidomics approach. Results are presented as mol %. (**c**) Total sphingomyelin, (**d**) sphingomyelin species, (**e**) total ceramide, (**f**) ceramide species. White bars, control; grey bars, fenofibrate. Data are shown as means ± SD; *n* = 4. *p < 0.05; **p < 0.01; ***p < 0.001 by two-tailed unpaired Student’s *t* test

We previously showed how fenofibrate prevented diabetes in NOD mice and how this was associated with increased islet sulfatide levels, as evaluated by immunohistochemistry [16]. To further evaluate the effect of fenofibrate on sulfatide levels, we employed an LC-MS approach. Female NOD mice were again treated with fenofibrate from the age of 3 weeks so as to precede the development of insulitis starting at age 4 weeks [23]. The LC-MS analysis showed an increase in the total amount of pancreatic sulfatide (*p* = 0.0145; Fig. 1a). This was entirely due to an increase in the amount of very-long-chain (≥20 carbons) C20:1, C24:0 and C24:1 sulfatide (*p* = 0.0017, *p* = 0.023 and *p* = 0.0008, respectively; Fig. 1b). Next, we used a shotgun lipidomics approach to evaluate the overall effect of fenofibrate on pancreatic lipids. The total amount of sphingomyelin was not altered (Fig. 1c), although we did observe a significant increase in very-long-chain C22:1 and C24:0 sphingomyelin (*p* = 0.027 and *p* = 0.004, respectively; Fig. 1d). The total amount of ceramide was also unaltered, but we found a changed composition of ceramide species, with a decrease in C16:0, C16:1 and C18:0 and an increase in C24:0 ceramide (*p* = 0.047, *p* = 0.006, *p* = 0.001 and *p* = 0.006, respectively; Fig. 1e, f).

### Increased levels of lysoGPLs

**Fig. 2:**
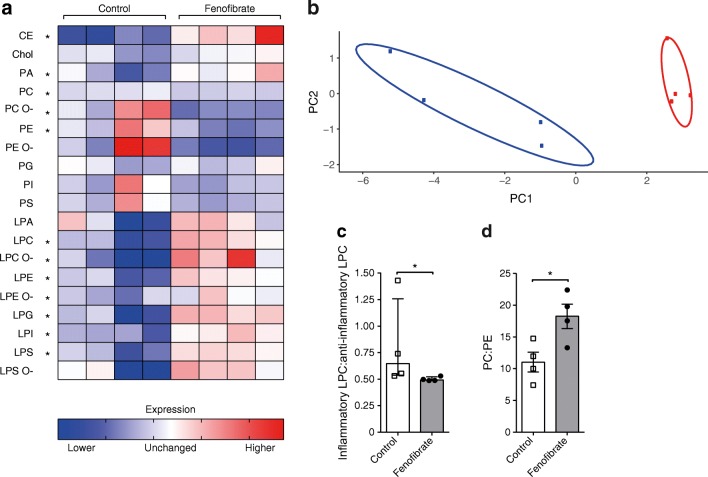
Fenofibrate alters the pancreatic lipidome. Pancreatic samples were analysed by MS. (**a**) Heatmap showing the changes in the lipid amounts of all non-sphingolipid species identified. Each column represents a separate mouse. Levels are normalised to the mean of all mice. Significantly altered lipid classes are marked by an asterisk. A false discovery rate of 5% was determined by the two-stage linear step-up procedure of Benjamini, Krieger and Yekutieli. (**b**) Principal component analysis of pancreas lipid composition. The contribution ratios of PC1 and PC2 (principal component 1 and 2) were 81.4% and 6.9%, respectively. Control is shown in blue and fenofibrate in red. (**c**) Ratio of saturated and monounsaturated LPC (inflammatory) to polyunsaturated LPC (anti-inflammatory). (**d**) Ratio of PC:PE. Data are medians with interquartile range, Mann–Whitney *U* test, in (**c**); means ± SD, unpaired two-tailed Student’s *t* test, in (**d**). *n* = 4. *p < 0.05. CE, cholesteryl ester; Chol, cholesterol; PA, phosphatidic acid; PG, phosphatidylglycerol; PI, phosphatidylinositol; PS, phosphatidylserine; LPA, lysophosphatic acid; LPE, lysophosphatidylethanolamine; LPG, lysophosphatidylglycerol; LPI, lysophosphatidylinositol; LPS, lysophosphatidylserine. The O- nomenclature indicates an ether lipid

Next, looking at the other lipid classes identified in our shotgun approach, we noticed a large variation within the control mice suggesting a loosely regulated pancreatic lipid metabolism in NOD mice (Fig. 2a). We found clear signs of degradation of GPLs, as evidenced by decreased levels of PC (*p* = 0.01), PC ether (*p* = 0.026) and PE (*p* = 0.031) and a corresponding increase in LPC (*p* = 0.005), LPC ether (*p* = 0.01), lysophosphatidylethanolamine (*p* = 0.013) and lysophosphatidylethanolamine ether (*p* = 0.04) in fenofibrate-treated mice (Fig. 2a). We also found increased levels of lysophosphatidylglycerol (*p* = 0.009), lysophosphatidylinositol (*p* = 0.002), lysophosphatidylserine (*p* = 0.007), phosphatidic acid (*p* = 0.034) and cholesteryl ester (*p* = 0.004). Furthermore, principal component analysis revealed a clear distinction between the pancreatic lipidome of control and fenofibrate-treated mice (Fig. 2b). LPC is the most abundant lysoGPL, with saturated and monounsaturated lipids known to promote inflammation while polyunsaturated lipids are anti-inflammatory [24]. We found increased levels of both inflammatory and anti-inflammatory LPCs with fenofibrate treatment. However, we found a significantly reduced ratio of inflammatory to anti-inflammatory LPCs, suggesting that fenofibrate promotes a more anti-inflammatory environment (*p* = 0.029; Fig. 2c). In addition, we observed an increased ratio of PC:PE with fenofibrate (*p* = 0.027; Fig. 2d), reflecting improved membrane integrity [25]. A list of all identified lipid species and their amounts can be found in the electronic supplementary material (ESM) Table 1.

### Fenofibrate prevents loss of pancreatic nerve fibres

**Fig. 3:**
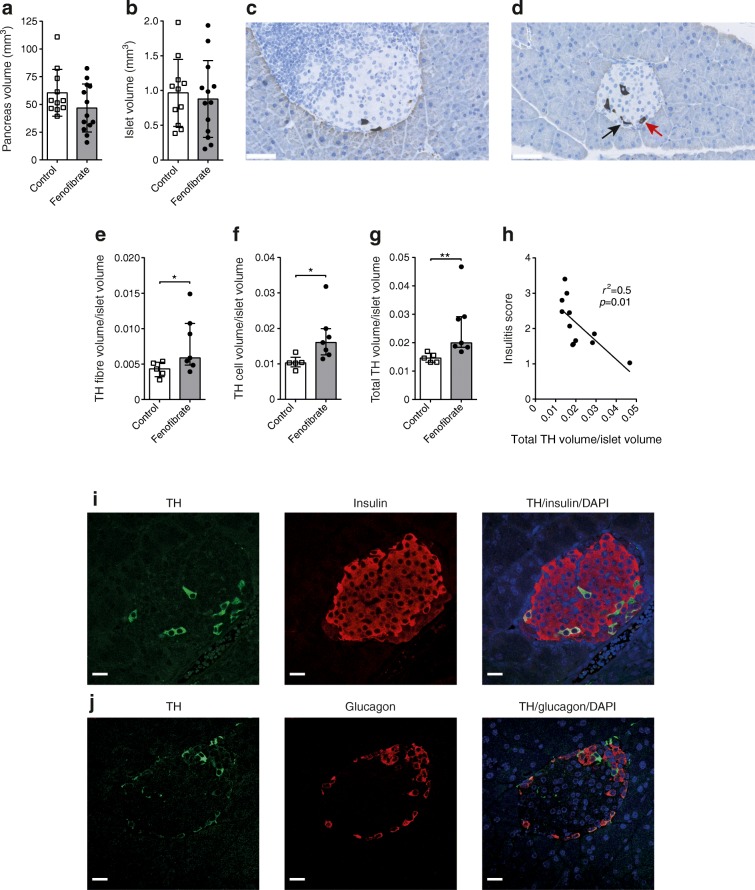
Higher TH volume in islets after fenofibrate treatment. (**a**) Pancreas and (**b**) islet volume in 13-week-old mice, as measured by stereology. Representative images showing TH staining in (**c**) control and (**d**) fenofibrate-treated 13-week old mice. Scale bars, 50 μm. Black arrow, nerve fibre; red arrow, TH-positive cell. (**e**) Nerve fibre volume/islet volume. (**f**) TH cell volume/islet volume. (**g**) Total TH volume (nerve fibre and cell)/islet volume. (**h**) Correlation between insulitis score and total TH volume/islet volume. (**i**) Image of a mouse islet with co-immunofluorescence staining of TH (green), insulin (red) and DAPI (blue). (**j**) Image of a mouse islet with co-immunofluorescence staining of TH (green), glucagon (red) and DAPI (blue). Scale bars, 20 μm. Data are means ± SD, unpaired two-tailed Student’s *t* test, in (**a**, **b**); medians with interquartile range, Mann–Whitney *U* test, in (**e–g**); linear regression in (**h**). Control, *n* = 11; fenofibrate, *n* = 13 in (**a**, **b**). Control, *n* = 5; fenofibrate, *n* = 7 in (**e–g**); *n* = 12 in (**h**). *p < 0.05, **p < 0.01

Next, we sought to evaluate if the altered lipidome would be reflected in altered pancreas morphology. Pancreases from another set of 13-week-old mice were analysed and showed that fenofibrate reduced insulitis to a similar degree as reported previously (ESM Fig. 1) [16]. Stereology examination showed no difference in pancreas or islet volume between control and fenofibrate-treated mice (Fig. 3a, b). C24:0 sulfatide is found in both islets and neuronal tissue, while C24:1 is primarily found in neuronal tissue [26]. We examined the neuronal volume in islets using TH, a sympathetic neuron marker [10] (Fig. 3c, d). We found a higher volume of islet sympathetic nerve fibres/islet volume (*p* = 0.048; Fig. 3e) and a higher volume of TH-expressing cells in islets/islet volume in fenofibrate-treated mice (*p* = 0.01; Fig. 3f). The total TH volume/islet volume was also higher in the fenofibrate-treated group (*p* = 0.005; Fig. 3g). There was an inverse correlation between insulitis score and total islet TH volume/islet volume (*p* = 0.01 and *r*^2^ = 0.5; Fig. 3h). To further understand the nature of these cells, we performed immunofluorescence to examine if the TH-positive cells co-expressed insulin or glucagon. The immunofluorescence revealed that 30% of TH-expressing cells also expressed insulin (TH-positive beta cells), while 31% also expressed glucagon (TH-positive alpha cells) (Fig. 3i, j).

There was no difference in pancreas or kidney weight between control and fenofibrate-treated mice, although we noted a reduced spleen weight and increased liver weight associated with liver hypertrophy in fenofibrate-treated mice (ESM Fig. 2), as described previously [27].

### Fenofibrate improves blood glucose homeostasis

**Fig. 4:**
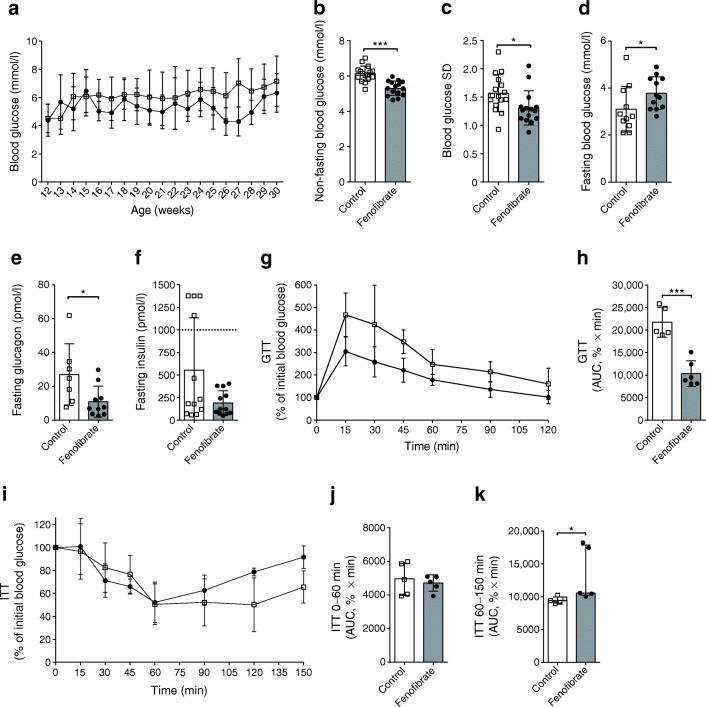
Fenofibrate improves blood glucose homeostasis. (**a**) Blood glucose levels were measured once a week under non-fasting conditions in healthy NOD mice from age 12 to 30 weeks. (**b**) Quantification of non-fasting blood glucose. (**c**) SD as a measurement of blood glucose stability. (**d–f**) Mice were fasted for 6 h before blood sampling and measurement of (**d**) blood glucose, (**e**) glucagon and (**f**) insulin levels. Mice with an insulin level above 1000 pmol/l (dashed line) were defined as hyperinsulinaemic. (**g**) GTTs were performed on healthy 13-week-old mice. Blood glucose was measured at the indicated times. (**h**) AUC calculation for evaluation of the GTT. (**i**) ITTs were performed on healthy 13-week-old mice and blood glucose was measured at the indicated times. (**j**) AUC calculation for the first 60 min (the decline period) of the ITT. (**k**) AUC calculation for time 60–150 min (the incline period) of the ITT. White squares, controls; black circles, fenofibrate-treated mice. Data are means ± SD, unpaired two-tailed Student’s *t* test, in (**a**–**j**); medians with interquartile range, Mann–Whitney *U* test, in (**k**). Control, *n* = 16; fenofibrate, *n* = 15 in (**a–c**). Control, *n* = 11; fenofibrate, *n* = 12 in (**d**). Control, *n* = 7; fenofibrate, *n* = 10 in (**e**). *n* = 12 in (**f**). Control, *n* = 5; fenofibrate. *n* = 6 in (**g**, **h**). *n* = 5 in (**i–k**). *p < 0.05. ***p < 0.001

Loss of pancreatic sympathetic neurons is known to reduce glucose tolerance in mice [28], so we investigated if the increased TH volume would be reflected in improved blood glucose homeostasis. Non-fasting blood glucose was measured weekly from age 12 to 30 weeks, and showed lower non-fasting blood glucose in fenofibrate-treated mice than in the control group (5.3 vs 6.1 mmol/l, *p* < 0.0001; Fig. 4a, b). Furthermore, fenofibrate treatment resulted in more stable blood glucose, as seen by the reduced SD (*p* = 0.024; Fig. 4c). Fasting blood glucose was increased in the fenofibrate group vs the control group (3.8 vs 3.1 mmol/l, *p* = 0.036; Fig. 4d). In addition, we found lower fasting glucagon levels in fenofibrate-treated mice (*p* = 0.019; Fig. 4e). Mice in the control group showed signs of fasting hyperinsulinaemia (fasting insulin above 1000 pmol/l: 4/12 mice [33%]). This was not observed in the fenofibrate-treated group (0/12 [0%], *p* = 0.03; Fig. 4f). Next, GTTs showed that fenofibrate-treated mice had improved glucose tolerance at age 13 weeks (*p* = 0.0002; Fig. 4g, h). Fenofibrate treatment did not induce any differences in insulin sensitivity, as evaluated with ITTs (*p* = 0.62; Fig. 4i, j). However, the ITT did show that fenofibrate-treated mice had an earlier return to normal glucose levels from insulin-induced hypoglycaemia (*p* = 0.016; Fig. 4k).

These effects were not the results of fenofibrate affecting body weight, although fenofibrate-treated mice did have increased water and food intake (ESM Fig. 3). Thus, fenofibrate improves glucose homeostasis.

## Discussion

A perturbed serum lipidome has been detected in children and NOD mice who later develop type 1 diabetes [29, 30]. However, there is a lack of studies looking at pancreas lipid composition in relation to type 1 diabetes. We have previously shown that individuals with newly diagnosed type 1 diabetes have abnormal islet sphingolipid metabolism. In this study, we sought to further investigate the effects of fenofibrate, a regulator of lipid metabolism that has previously been shown to prevent diabetes and inflammation in the pancreas of NOD mice [16]. Using MS, we found that fenofibrate treatment led to a specific increase in very-long-chain sphingolipids, predominantly C24. We observed no change in total ceramide levels, as the increase in C24 was associated with a corresponding decrease in C16 and C18. The altered chain length composition is critical, as the pro-apoptotic properties of ceramide are greatly dependent on the length of the lipid chain. C24 ceramide is an important component of myelin sheaths with essential roles in neuronal [31] and metabolic health [3, 32], whereas C16 ceramide promotes apoptosis [33] and is associated with mitochondrial dysfunction and insulin resistance [3, 34]. Hence, our results suggest that fenofibrate creates a more beneficial ceramide composition in the pancreas. The function of sulfatide is equally dependent on lipid chain length, with C16 regulating insulin secretion and proinsulin folding, while C24 is an immune regulator [35]. Furthermore, C24:1 sulfatide has been shown to induce transcription of the gene encoding indoleamine 2,3-dioxygenase 1, an important regulator of autoimmunity with reduced expression in individuals with newly diagnosed type 1 diabetes [36, 37]. The increase in C24:0 and C24:1 sulfatide is, thus, likely to create a more anti-inflammatory environment. This increase might be a direct explanation for the diabetes-protective effect of fenofibrate, as injections of C24:0 sulfatide have been shown to reduce diabetes incidence in NOD mice [35].

The progression into diabetes is associated with decreased levels of GPLs in NOD mice [29], suggesting that the reduced level of GPLs in fenofibrate-treated mice might be disadvantageous. On the other hand, we found increased amounts of lysoGPL, of which LPC is an inducer of insulin secretion [38]. The increased PC:PE ratio signals a changed membrane permeability, which could influence cytokine signalling; however, an increased ratio is also linked to endoplasmic reticulum stress [39]. A limitation of this study is the lack of a ‘normal’ pancreatic lipidome from non-diabetic mice, thus making the global biological effects of the observed changes hard to predict. It is difficult to find an appropriate control as different mouse strains have very different pancreatic lipidomes. A recent study found that the genetically closely related NOD-SCID mouse (which does not develop diabetes) has a very different pancreatic lipidome to the NOD mouse, making it a bad model with which to compare [40]. We are therefore at high risk of drawing different conclusions depending on which mouse strain we define as ‘normal’.

There is also a lack of studies looking at the pancreatic lipidome in humans, where studies have focused on blood lipid content. Human studies have, in this way, found decreased sphingolipids and LPC levels in serum [30, 41]. However, the serum lipidome is a poor predictor of the pancreatic lipidome [40]. Studies looking at the pancreatic lipidome across mouse strains and in humans are necessary to understand how the observed fenofibrate-induced changes in mice might be related to the development of type 1 diabetes.

Mouse pancreatic islets are highly innervated by sympathetic nerve fibres directly targeting alpha cells. In contrast, human islets are only sparsely innervated, with most axons contacting smooth muscle cells of blood vessels within the islet [42]. Despite these differences, islet sympathetic nerve fibres are lost in both NOD mice and individuals with type 1 diabetes [10, 11]. We observed a higher volume of TH-positive nerve fibres and cells within the pancreatic islets of fenofibrate-treated mice. The total TH volume was inversely correlated with the insulitis score, as previously described using the alternative sympathetic neuron marker neuropeptide Y [11]. The higher nerve fibre volume is likely not a result of neurogenesis, but rather of prevention of neural loss. This is in line with studies describing how fenofibrate prevents loss of nerves, as observed in diabetic retinopathy [43]. This might be explained by sulfatide being present in pericytes in the choroid layer, which regulate blood flow to the retina [44]. TH-positive alpha and beta cells have previously been described in mouse islets with a suggested role in beta cell development [45]. TH is a commonly used neuronal marker; however, the TH-positive cells in islets might be non-neural due to a lack of co-expression with other neuronal markers [45]. Our results suggest that the observed TH-positive cells in islets are a combination of alpha (31%) and beta (30%) cells. The nature of the remaining TH-positive cells is unknown; however, a subset of delta cells has been shown to express TH [46].

All animal species have relatively stable non-fasting blood glucose levels, also termed the glycaemic set point, and the set point of one species might be life-threatening for other species [47]. In this regard, it is interesting that fenofibrate seems to have caused a shift in the glycaemic set point, with lower non-fasting blood glucose and higher fasting blood glucose. This might be caused by the increased volume of islet nerve fibres or changes in alpha cell function, since these are known to influence the glycaemic set point [47, 48]. Fenofibrate-treated mice had a smaller difference in fasting to non-fasting blood glucose compared with controls, which might explain the increased blood glucose stability. This would be beneficial as blood glucose fluctuations are linked to an increased risk of diabetes-related complications [49]. The improved glucose tolerance further suggests overall improved blood glucose homeostasis, which is supported by signs of reduced islet hyperactivity as evidenced by a reduced degree of hyperinsulinaemia and reduced fasting glucagon levels. NOD mice are otherwise known to suffer from hyperinsulinaemia [50]. The results also suggest that fenofibrate might be beneficial in preventing hypoglycaemia, as fenofibrate-treated mice had an improved response to insulin-induced hypoglycaemia. Taken together, these results suggest that fenofibrate improves blood glucose homeostasis by improving islet function, as no change in insulin sensitivity was observed.

In conclusion, we have provided evidence that fenofibrate treatment leads to remodelling of the pancreatic lipidome, with increased levels of very-long-chain sphingolipids. This was associated with an increased volume of sympathetic nerve fibres in pancreatic islets and improved blood glucose homeostasis. These results, together with our previous finding of fenofibrate preventing and reversing diabetes in NOD mice, make fenofibrate a possible therapeutic approach. Clinical trials are needed to fully understand the therapeutic potential of fenofibrate.

## Electronic supplementary material

ESM table (XLSX 37 kb)

ESM figures (PDF 290 kb)

## Data Availability

All data generated and analysed during this study are included in this published article and its supplementary information files.

## Abbreviations

GPL: Glycerophospholipid
LPC: Lysophosphatidylcholine
PC: Phosphatidylcholine
PE: Phosphatidylethanolamine
TH: Tyrosine hydroxylase
