# Supplementary material for: Fenofibrate increases very-long-chain sphingolipids and improves blood glucose homeostasis in NOD mice
Source: Diabetologia. 2019 Aug 13;62(12):2262–72. doi: 10.1007/s00125-019-04973-z (PMC6861358; doi:10.1007/s00125-019-04973-z)
Supplement: Supplementary file 2 — (PDF 290 kb) [file 125_2019_4973_MOESM2_ESM.pdf]

Electronic Supplementary Material

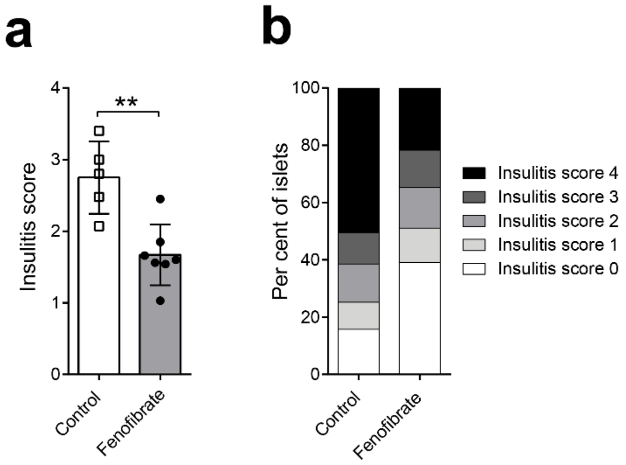

ESM Fig. 1 Fenofibrate reduces insulitis score. (**a**) Average insulitis score in 13-weeks-old NOD mice on a scale from 0 (no insulitis) to 4 (above 50% infiltration). (**b**) Percentage distribution of insulitis levels. Data are shown as means ± SD; Control n=5, Fenofibrate n=7; $^{**}p<0.01$ by two-tailed unpaired Student's t test

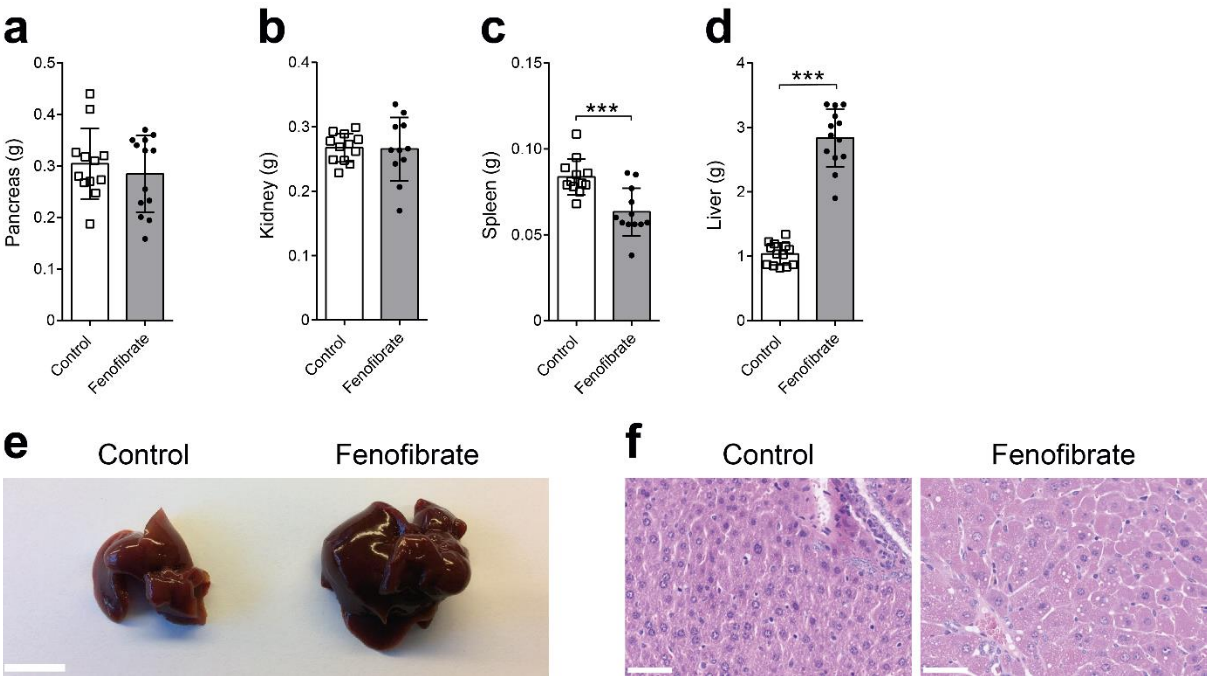

ESM Fig. 2 Fenofibrate induces liver hypertrophy. Organ weight was measured in 13-week-old mice, (**a**) Pancreas, (**b**) Kidney (right + left) and (**c**) Spleen. (**e**) Image showing the size of a standard liver as found in the two groups. Scale bar 1cm. (**f**) H&E stained liver showing enlarged cells in fenofibrate-treated mice. Scale bar 50µm. Data are shown as means ± SD. Control, n=12; fenofibrate, n=13 in (**a**). Control, n=12; fenofibrate, n=11 in (**b**). n=12 in (**c**). n=13 in (**d**). ***p<0.001 by two-tailed unpaired Student's t test

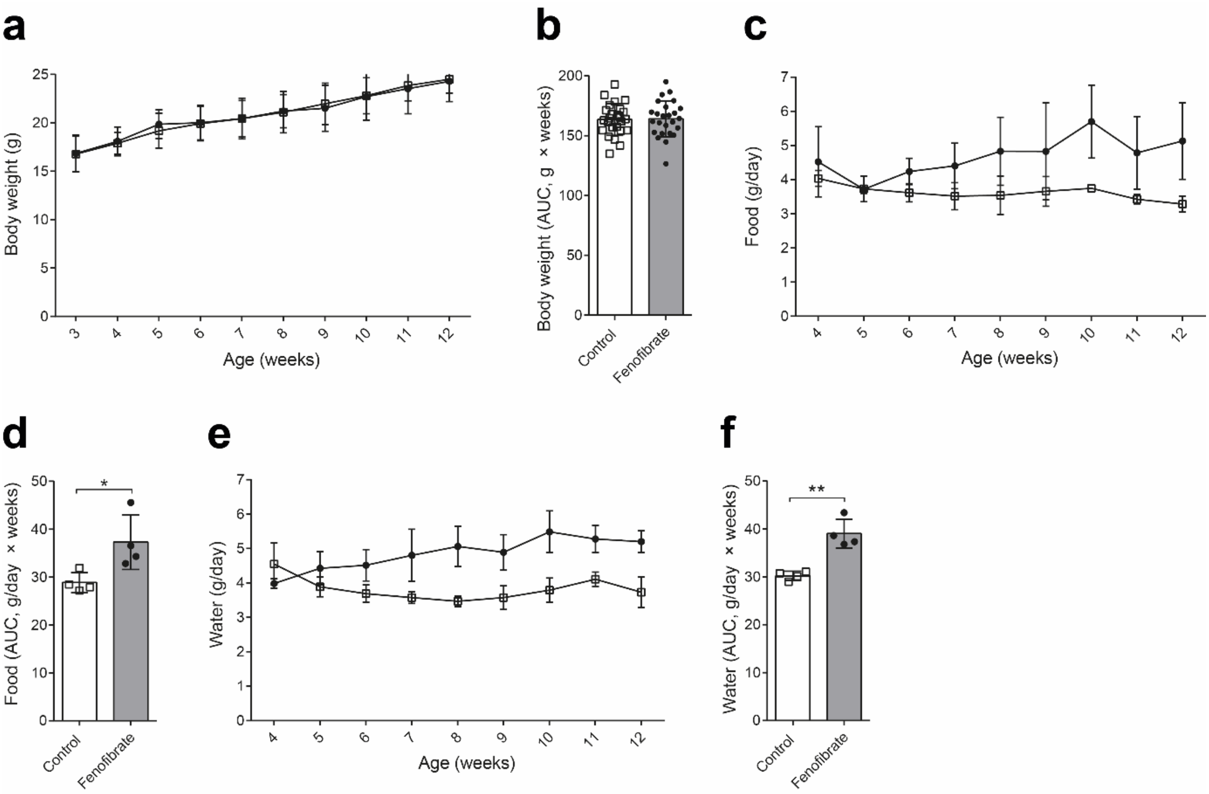

ESM Fig. 3 Fenofibrate increases food and water intake without affecting body weight. (**a**) Body weight as measured once a week in healthy NOD mice. (**b**) Body weight area under the curve (AUC) calculation. (**c**) Food intake was calculated per cage by weighing the food racks. (**d**) AUC calculation for food intake. (**e**) Water intake as calculated per cage weighing the water flasks. (**f**) AUC calculation for water intake. White squares represent control and black circles represent fenofibrate-treated mice. Data are shown as means ± SD. n=15 in (**a, b**). n=4 (**c, d, e, f**). *p<0.05. **p<0.01 by two-tailed unpaired Student's t test
